# A murine model of post-acute neurological sequelae following SARS-CoV-2 variant infection

**DOI:** 10.3389/fimmu.2024.1384516

**Published:** 2024-05-03

**Authors:** Ankita Singh, Awadalkareem Adam, Aditi, Bi-Hung Peng, Xiaoying Yu, Jing Zou, Vikram V. Kulkarni, Peter Kan, Wei Jiang, Pei-Yong Shi, Parimal Samir, Irma Cisneros, Tian Wang

**Affiliations:** ^1^ Department of Microbiology and Immunology, University of Texas Medical Branch, Galveston, TX, United States; ^2^ Department of Neuroscience, Cell Biology and Anatomy, University of Texas Medical Branch, Galveston, TX, United States; ^3^ Department of Preventive Medicine and Population Health, University of Texas Medical Branch, Galveston, TX, United States; ^4^ Department of Biochemistry and Molecular Biology, University of Texas Medical Branch, Galveston, TX, United States; ^5^ Department of Neurosurgery, University of Texas Medical Branch, Galveston, TX, United States; ^6^ Department of Microbiology and Immunology, Medical University of South Carolina, Charleston, SC, United States; ^7^ Department of Pathology, University of Texas Medical Branch, Galveston, TX, United States; ^8^ NeuroInfectious Diseases, University of Texas Medical Branch, Galveston, TX, United States; ^9^ Institute for Human Infections and Immunity, University of Texas Medical Branch, Galveston, TX, United States

**Keywords:** SARS-CoV-2 variant, post-acute sequelae, long COVID, inflammatory responses, CNS inflammation

## Abstract

Viral variant is one known risk factor associated with post-acute sequelae of COVID-19 (PASC), yet the pathogenesis is largely unknown. Here, we studied SARS-CoV-2 Delta variant-induced PASC in K18-hACE2 mice. The virus replicated productively, induced robust inflammatory responses in lung and brain tissues, and caused weight loss and mortality during the acute infection. Longitudinal behavior studies in surviving mice up to 4 months post-acute infection revealed persistent abnormalities in neuropsychiatric state and motor behaviors, while reflex and sensory functions recovered over time. In the brain, no detectable viral RNA and minimal residential immune cell activation was observed in the surviving mice post-acute infection. Transcriptome analysis revealed persistent activation of immune pathways, including humoral responses, complement, and phagocytosis, and gene expression levels associated with ataxia telangiectasia, impaired cognitive function and memory recall, and neuronal dysfunction and degeneration. Furthermore, surviving mice maintained potent systemic T helper 1 prone cellular immune responses and strong sera neutralizing antibodies against Delta and Omicron variants months post-acute infection. Overall, our findings suggest that infection in K18-hACE2 mice recapitulates the persistent clinical symptoms reported in long-COVID patients and provides new insights into the role of systemic and brain residential immune factors in PASC pathogenesis.

## 1 Introduction

Acute COVID-19 infection typically lasts 4 weeks from symptomatic onset and results in diverse clinical outcome ranging from mild to severe pneumonia, life-threatening multiorgan failure, and death (1, 2). An estimated 30% to 50% of COVID-19 survivors suffer from a post-viral syndrome known as long-COVID [post-acute sequelae of COVID-19 (PASC)], which encompasses ongoing persistent symptoms and complications beyond the initial 4 weeks of infection (3, 4). The key features of long-COVID include neurological symptoms, such as fatigue, cognitive dysfunction (brain fog, memory loss, and attention disorder), and sleep disturbances (5–7). Psychiatric manifestations (anxiety and depression) are also common (8, 9). Although the severity of acute infection is considered as one major risk factor for developing PASC (10, 11), increasing evidence suggests that long-COVID also occurs in people with non-symptomatic or non-hospitalized status during acute infection (12, 13). PASC has posed a significant threat to the global healthcare system. Nevertheless, our current understanding of its underlying mechanisms is limited.

Acute SARS-CoV-2 infection has been studied in various animal models, including hamsters, ferrets, non-human primates (NHPs), rats, and mice (14, 15). Mice are easier to work with, and most amenable to immunological manipulation. Angiotensin-converting enzyme 2 (ACE2) is the cell entry receptor for SARS-CoV-2 (16), and mouse ACE2 shows key differences from human ACE2 (hACE2); thus, wild-type mice present challenges for infection with human SARS-CoV-2 variants. To overcome this challenge, delivery of adeno-associated virus (AAV)-mediated expression of hACE2 into the respiratory tract or use of the mouse-adapted SARS-CoV-2 (SARS-CoV-2 MA strain or CMA strain), which incorporates key mutations that allows the virus to utilize mouse ACE2 for entry into cells in immunocompetent mice, results in a productive infection with mild acute respiratory distress syndrome (17–19). K18-hACE2 transgenic mice express the hACE2 protein under the human keratin 18 (K18) promoter, which confers efficient transgene expression in airway epithelial cells. Acute SARS-CoV-2 infection in K18-hACE2 transgenic mice induces weight loss, interstitial pneumonitis, encephalitis, and death (20–23). In this study, to investigate SARS-CoV-2 variant-induced PASC, we infected K18-hACE2 mice with the Delta variant and performed longitudinal behavior analysis for up to 4 months post-acute infection. Mice surviving acute Delta variant infection displayed persistent abnormalities in neuropsychiatric state and motor behavior post-acute infection. Although surviving mice developed and maintained potent Th1-prone cellular and antibody responses in the periphery, transcriptome analysis suggested persistent activation of immune pathways, cognitive dysfunction and neuronal dysfunction in the CNS for months post-acute infection.

## 2 Materials and methods

Viruses: SARS-CoV-2 Delta and Omicron B.A.2 strains were obtained from the World Reference Center for Emerging Viruses and Arboviruses (WRCEVA) at the University of Texas Medical Branch (UTMB) and were amplified twice in Vero E6 cells as described previously (24).

### 2.1 SARS-CoV-2 infection in mice and ethics statement

Six- to 8-week-old K18-hACE2 mice (Jackson Lab, stock #034860) were bred and maintained at UTMB animal facility. All animal experiments were approved by the Institutional Animal Care and Use Committee (IACUC # 1412070) at UTMB. In order to study virus-induced PASC, mice were infected intranasally (i.n.) with sublethal doses [600 and 800 plaque-forming units (PFU) of SARS-CoV-2 Delta or Omicron B.A.2 strain, respectively. based on Singh et al. unpublished data]. Infected mice were monitored twice daily for morbidity and mortality. In some experiments, on 3 days, 6 days, 7 days, 8 days, 9 days, 1 month, 2 months, and 4 months post-infection (pi), mice were transcardially perfused with PBS to remove blood. Hemi brains and left lungs were collected in 4% paraformaldehyde for histopathology and immunofluorescence staining studies. The right, superior, and middle lobes of mouse lung, hemi brains, kidney, and liver tissues were collected in Trizol for RNA extraction used for viral RNA and cytokine analysis. The inferior lobes of right lung and hemi brains (in some experiments) were homogenized in 1 mL of PBS and viral titers in the tissue homogenates were further determined by plaque assays as PFU per lobe of lung (19).

### 2.2 Quantitative PCR

The sequences of the primer sets and PCR reaction conditions were described previously (25–27) and in **Supplementary Table S1**. Tissues were resuspended in TRIzol for RNA extraction according to the manufacturer’s instructions (Thermo Fisher Scientific). RNA concentration and purity were determined by using WPA Biowave DNA Spectrophotometer. Complementary (c) DNA was then synthesized by using a qScript cDNA synthesis kit (Bio-Rad). Expression of SARS-CoV-2 S gene and mouse inflammatory cytokine and chemokine genes (IL-1β, IL-6, TNF-α, CCL2, CCL5, CCL7, CXCL10, and CCL11) were measured by quantitative PCR (Q-PCR) using the CFX96 real-time PCR system (Bio-Rad). PCR cycling conditions were as follows: 95°C for 3 min, 45 cycles of 95°C for 15 s, and 60°C for 1 min. Gene expression was calculated using the formula 2^−[Ct(target gene)−Ct(β-actin)]^ as described before (28).

### 2.3 RNA-seq, gene set enrichment analysis, and Cytoscape analysis

RNA was extracted from brain tissues as described above and 1–3 µg of RNA of each sample was used for RNA-seq analysis. The RNA quality was verified by the Next-Generation Sequence (NGS) core laboratory using a Nanodrop ND-1000 spectrophotometer (Thermo Fisher Scientific) and an Agilent Bioanalyzer 2100 (Agilent Technologies, Santa Clara, CA). PolyA+ RNA was purified from ~100 ng of total RNA and sequencing libraries were prepared with the NEBNext Ultra II RNA library kit (New England Biolabs) following the manufacturer’s protocol. Libraries were pooled and sequenced on an Illumina NextSeq 550 High Output flow cell with a paired-end 75 base protocol. Pathway enrichment analysis was performed using gene set enrichment analysis (GSEA) version 3.0 (29, 30). Specifically, analysis parameters were set to 2,000 gene set permutation and gene set size limit 15–500. Primary gene sets investigated were obtained from the David Bader lab (http://download.baderlab.org/EM_Genesets/). GSEA FDR *Q* < 0.05 cutoff was applied to examine enriched gene sets in our dataset. Cytoscape Enrichment map tool was used to visualize results from the GSEA. GSEA output files were uploaded in the Enrichment Map app of Cytoscape and FDR *Q* value cutoff was set to 0.01. The AutoAnnotate Cytoscape app was used to define the clusters automatically. The RNA-seq data in this study were deposited in NCBI’s Gene Expression Omnibus (GEO Series accession number GSE260625). The sequence can be found at https://www.ncbi.nlm.nih.gov/geo/query/acc.cgi?acc=GSE260625.

### 2.4 Plaque assay

Vero E6 cells were seeded in six-well plates and incubated at 37°C. Tissue homogenates were serially diluted (10-fold) in DMEM with 2% FBS and 0.2 mL was used to infect cells at 37°C for 1 h. After incubation, samples were overlaid with MEM (Gibco) with 8% FBS and 1.6% agarose (Promega). After 48 h, plates were stained with 0.05% neutral red (Sigma-Aldrich) and plaques were counted to calculate virus titers expressed as PFU/mL (24, 31).

### 2.5 Histopathology studies

As previously described with some modifications (19), left lung and hemi brain tissues were collected and fixed in 4% paraformaldehyde for a minimum of 3 days at 4°C. The fixed tissues were then transferred and submerged in 10% buffered formalin (Fisher, Waltham, MA). Preparation of paraffin-embedded tissue blocks followed by cutting 10-µm tissue sections and hematoxylin and eosin (H&E) staining were performed by the Histopathology Laboratory Core at UTMB. Unstained tissue sections were also prepared for immunofluorescence staining.

### 2.6 Behavioral studies

At 1, 2, 3, and 4 months pi, all surviving animals underwent neurological assessments using a modified SmithKline Beecham, Harwell, Imperial College, Royal London Hospital phenotype assessment (SHIRPA) protocol (32, 33). Mice were also weighed at the above time points to confirm their growth. For the modified SHIRPA assessment, each mouse was placed in a transparent cylindrical viewing jar and observed for 5 min. Observations in body position, spontaneous activity, respiration rate, tremor, and defecation were noted. Subsequently, the mouse was transferred to an open field arena at which time transfer arousal and gait were noted. Following transfer, the mice were allowed to freely move around the open field arena for 30 s and the number of times that all four limbs crossed into new quadrants was counted to evaluate locomotor activity. For the next 5 min, gait, eye opening, piloerection, pelvic elevation, tail position, and touch escape were observed in the open field arena. Furthermore, tail lifting was performed to evaluate trunk curl and visual placing followed by assessment of reach touch, grip strength, whisker response, palpebral reflex, and ear twitch above arena. Additional behaviors of each mouse, such as fear, biting, irritability, and aggression, were observed throughout the procedure. Based on the observation, scores were provided (0, 1, 2, or 3); 2 was considered normal behavior, and any score outside of 2 was considered abnormal behavior. Each parameter assessed by SHIRPA was grouped into five functional categories (see **Table 1**). To measure motor coordination, a parallel rod floor test was performed. Briefly, mice were placed in the center of the cage that was covered with horizontal rods for 2 min. Foot/paw slips were counted and recorded manually.

**Table 1:** Modified SHIRPA assessment.

1. Neuropsychiatric state	Spontaneous activity, transfer arousal, touch escape, positional passivity, biting, fear, irritability, and aggression
2. Motor behavior	Body position, tremor, locomotor activity, pelvic elevation, tail elevation, gait, trunk curl, and limb grasping
3. Autonomic function	Respiration rate, piloerection, and heart rate
4. Muscle tone and strength	Grip strength, body tone, limb tone, and abdominal tone
5. Reflex and sensory functions	Visual placement, pinna reflex, toe pinch, righting reflex, palpebral reflex, whisker response, reach touch, and ear twitch

### 2.7 Immunofluorescence staining

Formalin-fixed, paraffin-embedded tissue blocks were cut into 10-μm sections, which were deparaffinized with xylene and rehydrated with ethanol/water. To detect Iba1 and GFAP expression, tissue sections were exposed to anti-Iba1 rabbit antibody (FUJIFILM Wacko, 013-27691, 1:300 dilution) and anti-GFAP mouse antibody (Sigma, G3893, 1:300 dilution) overnight at room temperature, respectively. After washing with phosphate-buffered saline (PBS), slides were exposed to goat anti-rabbit Alexa Fluor Plus 555 (Thermo Fisher Scientific, A32732, 1:1,000 dilution) and goat anti-mouse Alexa Fluor 488 (Thermo Fisher Scientific, A32723, 1:1,000 dilution) secondary antibodies for 1 h at room temperature. For nuclei staining, we used 4′,6′-diamidino-2-phenylindole (DAPI) (D9542, Sigma). Slides were washed with PBS and mounted with ProLong Gold Antifade (Thermo Fisher Scientific, P36930). Images were captured using an Olympus BX53 microscope.

### 2.8 Antibody and SARS-CoV-2 spike (S) protein ELISA

As described previously (24, 31), ELISA plates were coated with 100 ng/well recombinant SARS-CoV-2 RBD protein (RayBiotech) overnight at 4°C. The plates were washed twice with PBS containing 0.05% Tween-20 (PBS-T) and then blocked with 8% FBS for 1.5 h. Sera were diluted 1:100 in blocking buffer and were added for 1 h at 37°C. Plates were washed five times with PBS-T. Goat anti-mouse IgG (Sigma) coupled to horseradish peroxidase (HRP) or alkaline phosphatase was added at a 1:2,000 dilution for 1 h at 37°C. This was followed by adding TMB (3,3,5,5′-tetramethylbenzidine) peroxidase substrate (Thermo Fisher Scientific) for approximately 10 min. The reactions were stopped with 1 M sulfuric acid, and the intensity was read at an absorbance of 450 nm. For SARS-CoV-2 S protein ELISA, plates were coated with 50 ng/well (5 µg/mL in coating buffer) of diluted capture antibody (Thermo Fisher Scientific, USA) overnight at 4°C. The plates were washed twice with PBS-Tween (PBS-T) and then blocked with 8% FBS for 1.5 h. Standard (recombinant SARS-CoV-2 S protein, Sino Biological, USA) was diluted in blocking buffer, and the brain suspension was added for 1 h at 37°C. Plates were washed five times with PBS-T. SARS-CoV-2 S1 protein primary antibody (Thermo Fisher Scientific) at 1 µg/mL was added for 1 h at 37°C. Plates were again washed five times with PBS-T. Goat anti-rabbit IgG (Thermo Fisher Scientific) coupled to horseradish peroxidase (HRP) was added at a 1:2,000 dilution for 1 h at 37°C, followed by adding TMB (Thermo Fisher Scientific) for 30 min. The reactions were stopped by 1 M sulfuric acid, and the intensity was read at an absorbance of 450 nm.

### 2.9 IFN-γ ELISPOT

As described earlier (24), Millipore ELISPOT plates (Millipore Ltd.) were coated with anti-mouse IFN-γ capture Ab at 1:100 dilution (Cellular Technology Ltd) at 4°C overnight. Splenocytes were stimulated with SARS-CoV-2 S peptide pools (2 μg/mL, Miltenyi Biotec) for 24 h at 37°C. Cells were stimulated with anti-CD3 (1 μg/mL, e-Biosciences) or medium alone, as controls. This was followed by incubation with biotin-conjugated anti-IFN-γ at 1:100 dilution (Cellular Technology Ltd.) for 2 h at room temperature, followed by incubation with alkaline phosphatase-conjugated streptavidin for 30 min. The plates were washed and scanned using an ImmunoSpot 6.0 analyzer and analyzed by ImmunoSpot software to determine the spot-forming cells (SFCs) per 10^6^ splenocytes.

### 2.10 Intracellular cytokine staining

Splenocytes were incubated with SARS-CoV-2 S peptide pools (1 μg/mL, Miltenyi Biotec) for 6 h in the presence of GolgiPlug (BD Bioscience). Cells were harvested and stained with antibodies for CD3, CD4, or CD8, fixed in 2% paraformaldehyde, and permeabilized with 0.5% saponin before adding anti-IFN-γ (Thermo Fisher Scientific) (24, 31). Samples were acquired by a C6 Flow Cytometer instrument. Dead cells were excluded based on forward and side light scatter. Data were analyzed with a CFlow Plus Flow Cytometer (BD Biosciences).

### 2.11 Fluorescent focus reduction neutralization test

Neutralization titers of mice sera were measured by a fluorescent focus reduction neutralization test (FFRNT) using the mNG reporter SARS-CoV-2 as previously reported with some modifications (24, 34). Briefly, Vero E6 cells (2.5 × 10^4^) were seeded in each well of a black μCLEAR flat-bottom 96-well plate (Greiner Bio-one™). The cells were incubated overnight at 37°C with 5% CO_2_. On the following day, each serum was twofold serially diluted in the culture medium with the first dilution of 1:20. Each serum was tested in duplicate. The diluted serum was incubated with 100–150 fluorescent focus units (FFU) of mNG Delta and BA.2 SARS-CoV-2 at 37°C for 1 h (final dilution range of 1:20 to 1:20,480), respectively. After that, the serum–virus mixtures were inoculated onto the pre-seeded Vero E6 cell monolayer in 96-well plates. After 1 h of infection, the inoculum was removed and 100 μL of overlay medium (DMEM supplemented with 0.8% methylcellulose, 2% FBS, and 1% P/S) was added to each well. After incubating the plates at 37°C for 16 h, raw images of mNG fluorescent foci were acquired using CytationTM 7 (BioTek) armed with 2.5× FL Zeiss objective with a wide field of view and processed using the software settings [GFP (469,525) threshold 4,000, object selection size 50–1,000 µm]. The foci in each well were counted and normalized to the non-serum-treated controls (set as 100%) to calculate the relative infectivity. The neutralizing titer 50 (NT_50_) was calculated manually as the highest dilution of the serum sample that prevents at least 50% fluorescence foci formation in infected cells. A titer is calculated for each of the two replicates of a sample and the geometric mean titer (GMT) of the two is reported as the final sample titer.

### 2.12 Statistical analysis

Survival curve comparison was performed using GraphPad Prism software 9.4.1, which uses the log-rank test. Values for viral load, cytokine production, antibody titers, and T-cell response experiments were compared using Prism software statistical analysis and were presented as means ± SEM. *P* values of these experiments were calculated with a non-paired Student’s ***t***-test. Parameters of behavior changes at month 1 and results of parallel rod test were compared using Student’s ***t***-test. For the categorical, longitudinal measures of each parameter in modified SHIRPA testing, we considered a score of 2 as normal activity and the other scores (0, 1, and 3) as abnormal activity. For selected parameters, changes were presented over time in a stacked bar chart with Sankey-style overlays using SAS version 9.4 (SAS Inc., Cary, NC). All tests were two-sided with a significance level of 0.05.

## 3 Results

### 3.1 SARS-CoV-2 variant replicated in brain and lung tissues and induced inflammatory responses in both tissues during the acute infection in K18-hACE2 mice

**Figure 1:**
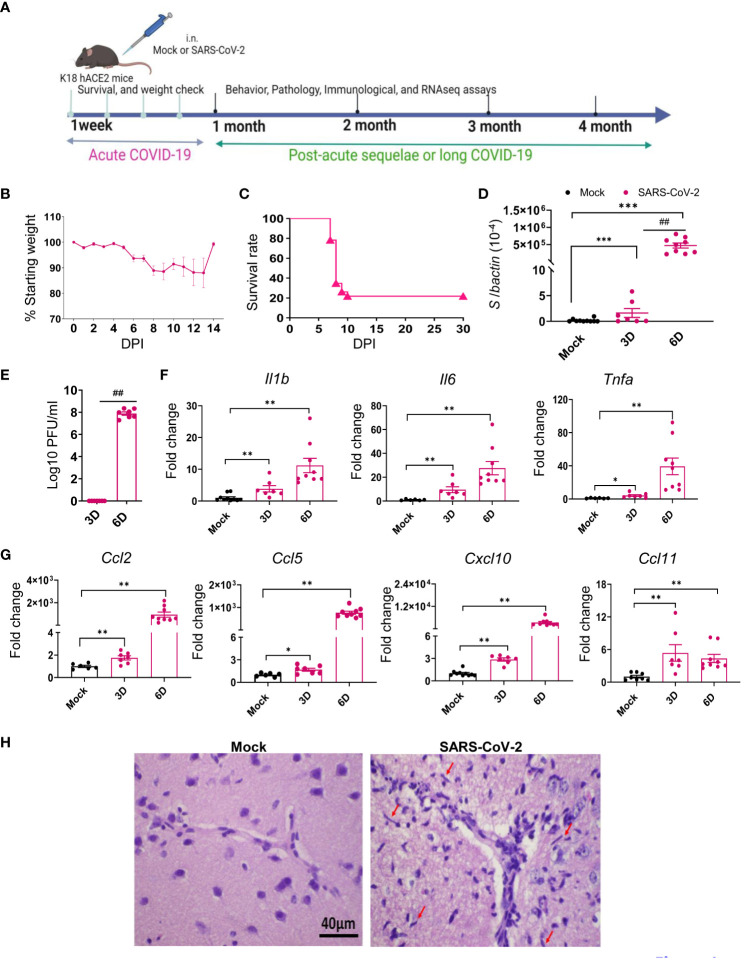
SARS-CoV-2 Delta variant replicated and induced inflammatory responses in brain tissues during acute infection in K18-hACE2 mice. Six- to eight-week-old K18-hACE2 mice were i.n. infected with a sublethal dose of SARS-CoV-2 Delta variant strain (or mock infected) and monitored daily for morbidity and mortality. **(A)** Study design. **(B)** Mouse weight loss. Weight loss is indicated by percentage using the weight on the day of infection as 100%. *n* = 23. **(C)** Survival rate. **(D, E)** SARS-CoV-2 viral loads in brain were measured by Q-PCR **(D)** and plaque assay **(E)** at indicated days **(D)** pi. **(F, G)** Cytokine **(F)** and chemokine **(G)** expression levels in the brains were measured by Q-PCR. Data are presented as the fold increase compared to mock-infected mice (means ± SEM). *n* = 7 to 10. **(H)** Histopathology of brains of Delta variant-infected mice revealed viral encephalitis with perivascular infiltrations and microglial activation (arrows) in the cortex, but not in mock-infected mice ***p < 0.001, **p < 0.01, or *p < 0.05 compared to mock. ^##^ *p* < 0.01 compared to 3D.

**Figure 2:**
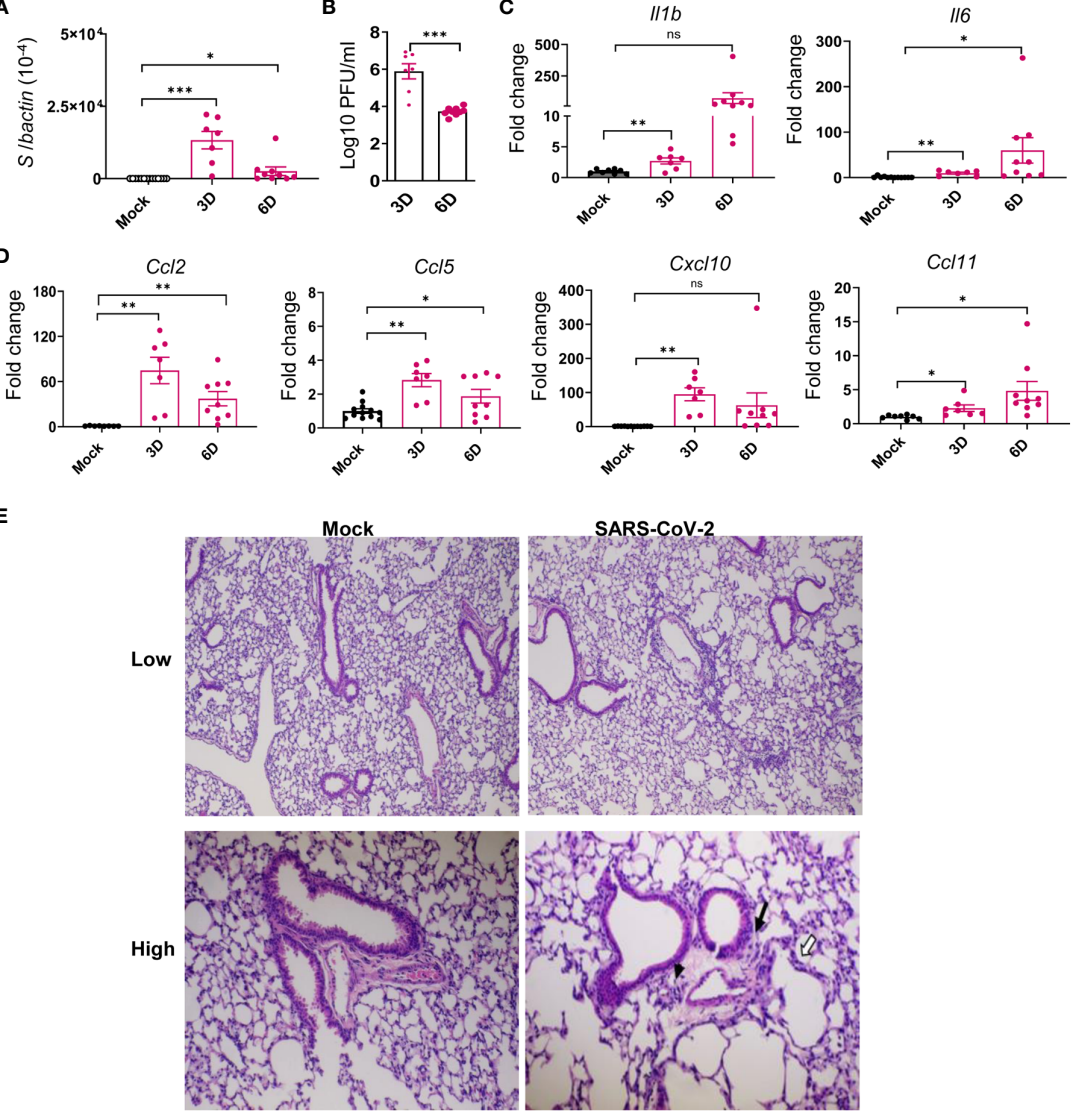
SARS-CoV-2 Delta variant replicated in the lung tissues and induced inflammatory responses in K18-hACE2 mice during acute infection. Six- to eight-week-old K18-hACE2 mice were i.n. infected with a sublethal dose of SARS-CoV-2 Delta variant strain or mock infected. Viral loads in lung tissues were measured by Q-PCR **(A)** and plaque assays **(B)** at indicated days **(D)** pi. **(C, D)** Cytokine **(C)** and chemokine **(D)** expression levels in the lung were measured by Q-PCR. Data are presented as the fold increase compared to mock-infected mice (means ± SEM). *n* = 7 to 9. **(E)** Histopathology of Delta variant-infected (right panels) or mock-infected (left panels) lungs. Low-power views of representative areas (top panels) from each group show moderate inflammation in the infected mice. At higher-power views (low panels), mononuclear cell infiltrations are observed in peribronchiolar (black arrow) and perivascular (arrowhead) areas as well as in the alveolar septa (white arrows). Bar = 200 μm for top panels; Bar = 80 μm for low panels. ns, not statistically significant ***p < 0.001, **p < 0.01, or *p < 0.05 compared to mock.

Several reports suggest that the frequencies of PASC symptoms increased with SARS-CoV-2 variants, in particular, with the pre-Omicron variant compared to the original prototype virus infection (3, 35–37). Thus, to investigate the mechanisms of SARS-CoV-2-induced PASC, we infected K18-hACE2 mice with the Delta variant. Viral load analysis, survival/weight changes monitoring, immunological and histopathology studies, and behavior assessment were performed at both acute infection and at 1 to 4 months post-acute COVID infection (**Figure 1A**). Initially, 6-to 8-week-old K18-hACE2 mice were intranasally (i.n.) inoculated with a sublethal dose of SARS-CoV-2 Delta variant strain and monitored daily for morbidity and mortality. Infected mice exhibited weight loss starting on day 6 pi and succumbed to infection as early as day 7. Approximately 22% of mice infected with the Delta variant survived the 4-week pi interval (**Figures 1B, C**). In the brain, viral RNA but not infectious virus was detected at day 3. Viral loads increased significantly and reached to the peak at day 6. Viral RNA levels were continuously detectable at days 7, 8, and 9 pi (**Figures 1D, E**, **Supplementary Figure 1A**). As in the most severe clinical cases, the prognosis can be worsened by the hyperproduction of proinflammatory cytokines (38, 39); we next measured expression levels of proinflammatory cytokines and chemokines. At days 3 and 6, proinflammatory cytokines, including IL-1β, IL-6, and TNF-α, and chemokines, such as CCL2, CCL5, CXCL10, and CCL11, were induced in the brains of infected mice (**Figures 1F, G**). Histopathology analysis was also performed to confirm these findings and revealed viral encephalitis with perivascular infiltrations and microglial activation in the cortex of infected mice, but not in mock-infected mice, which together suggest neuroinflammation induction in the Delta variant-infected mice (**Figure 1H**). As lung is the primary site of SARS-CoV-2 infection, we next measured viral loads in the lung and noted infection was high at day 3 but diminished at day 6 (**Figures 2A, B**). Viral RNA remained detectable in the lungs at days 7, 8, and 9 pi (**Supplementary Figure 1B**). Proinflammatory cytokines and chemokines, including IL-1β, IL-6, CCL2, CCL5, CXCL10, and CCL11, were triggered in the lungs of infected mice compared to the mock group (**Figures 2C, D**). Lung pathology study showed mononuclear cell infiltration in peribronchiolar and perivascular areas as well as in the alveolar septa of the infected mice, but not in the mock group (**Figure 2E**). Overall, these results suggest that the SARS-CoV-2 Delta variant replicated in lung and brain tissues and triggered inflammation during the acute infection phase in K18-hACE2 mice.

### 3.2 SARS-CoV-2 Delta variant-infected mice displayed neurological behavior changes months post-acute infection

Based on National Institute for Health and Care Excellence guidelines, PASC is displayed at 4 weeks or more after the start of acute COVID-19 infection ^3^. Here, we did not detect infectious virus (data not shown) or viral RNAs (**Supplementary Figures 1A, B**) at 1 and 4 months pi in the brain and lung tissues. No significant levels of viral S1 protein were detected in the brain tissues beyond 1 month, which together indicate viral clearance at the post-acute phase (**Supplementary Figure 1C**). To further understand the effects of SARS-CoV-2 infection in PASC pathogenesis, viral loads in several other periphery tissues, including liver, kidney, and blood, were also measured during acute and post-acute infection. No detectable viral RNA was found in these tissues except at day 6 in the kidneys (**Supplementary Figures 1D–F**). Histopathological analysis revealed no changes in the various regions of brain in the Delta variant-infected mice at 1 or 4 months pi compared to the mock group (**Supplementary Figure 2A**). In the lung, there were notably increased levels of CCL7 and CXCL-10 at 1 month, and increased levels of IL-6, CCL5, CXCL10, and CCL11 at 4 months in the infected mice compared to the mock group (**Supplementary Figure 2B**), though no changes in the levels of IL-1β, TNF-α, and CCL2 were observed (data not shown). Thus, viral infection was cleared in the periphery and CNS tissues post-acute infection, and this was accompanied by mild and minimal local inflammatory responses in the lung and brain tissues, respectively.

**Figure 3:**
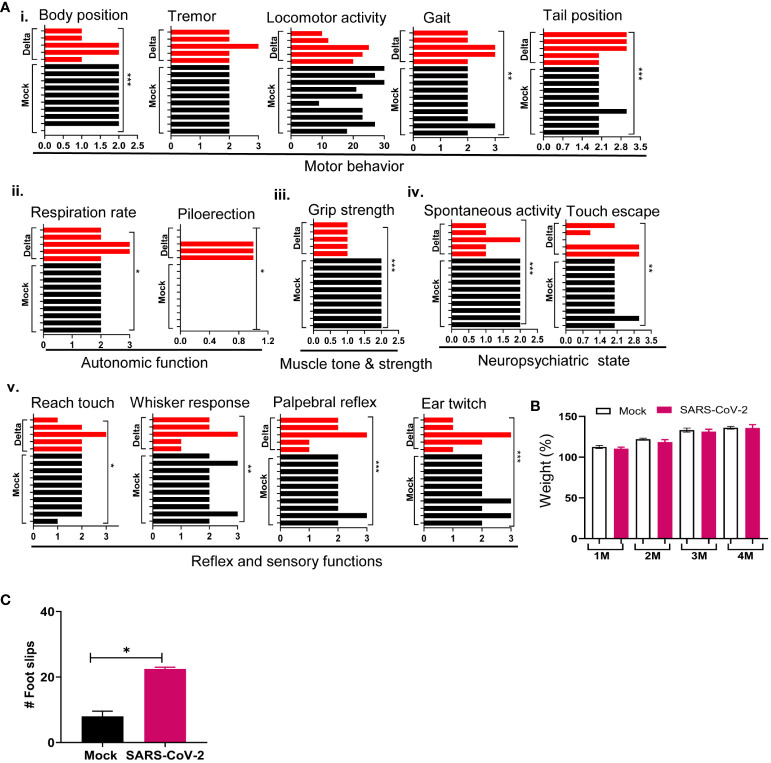
SARS-CoV-2 Delta variant induces behaviors changes post-acute infection. K18-hACE2 mice were infected with a sublethal dose of SARS-CoV-2 Delta variant, or PBS (mock). At 1 month pi or after, surviving mice (*n* = 10 for mock and *n* = 5 for SARS-CoV-2 Delta variant-infected mice) were assessed for behavior changes via SHIRPA **(A)**, weight changes **(B)**, and a parallel rod floor test **(C)**. **(A)** At 1 month pi, SARS-CoV-2 variant-infected mice showed impaired performance in the SHIRPA assessment. (i) Motor behavior; (ii) autonomic function; (iii) muscle tone and strength; (iv) neuropsychiatric state; and (v) Reflex and sensory functions. The *y*-axis represents individual mouse for the mock (*n* = 10) and Delta variant-infected groups (*n* = 5). **(B)** Weight changes during the 4-month pi interval presented as percentage using the weight on the day of infection as 100%. **(C)** Parallel rod floor test. At 3 months pi, surviving mice were placed in the center of the cage coated with horizontal rods for 2 min. Foot/paw slips were counted **(C)**. ***p < 0.001, **p < 0.01, or *p < 0.05 compared to the mock group.

To assess the infection impact on animals, behavior tests were performed in the surviving mice at 1 month pi using a modified Smith-Kline Beecham, Harwell, Imperial College, Royal London Hospital, phenotype assessment (SHIRPA) protocol (32, 33). Mock-infected mice were used as controls. The assay involves a battery of semi-quantitative tests for general health and sensory function, baseline behaviors, and neurological reflexes. The individual parameters assessed by SHIRPA were grouped into five functional categories (**Table 1**): (1) Motor behavior test includes body position, tremor, locomotor activity, pelvic elevation, tail elevation, gait, trunk curl, and limb grasping; (2) Autonomic function test includes respiration rate, palpebral closure, and piloerection; (3) Muscle tone and strength includes grip strength, body tone, and limb tone; (4) Neuropsychiatric state includes spontaneous activity, transfer arousal, touch escape, positional passivity, biting, fear, irritability, and aggression; and (5) Reflex and sensory functions include parameters such as visual placement, toe pinch, and righting reflex (40). The SHIRPA assay results showed that mice surviving acute infection with SARS-CoV-2 Delta variant displayed abnormalities mainly in neuropsychiatric state, motor behavior, autonomic function, and reflex and sensory function, compared to the mock group (**Figure 3A**). Weight loss was not noted in the surviving mice at 1 month pi, nor was it detected during the rest of the 4-month pi interval (**Figure 3B**). Furthermore, the Delta variant-infected mice showed higher number of foot slips compared to the mock group at 3 months pi in a parallel rod test, which indicates ataxia in the infected mice (41) (**Figure 3C**).

**Figure 4:**
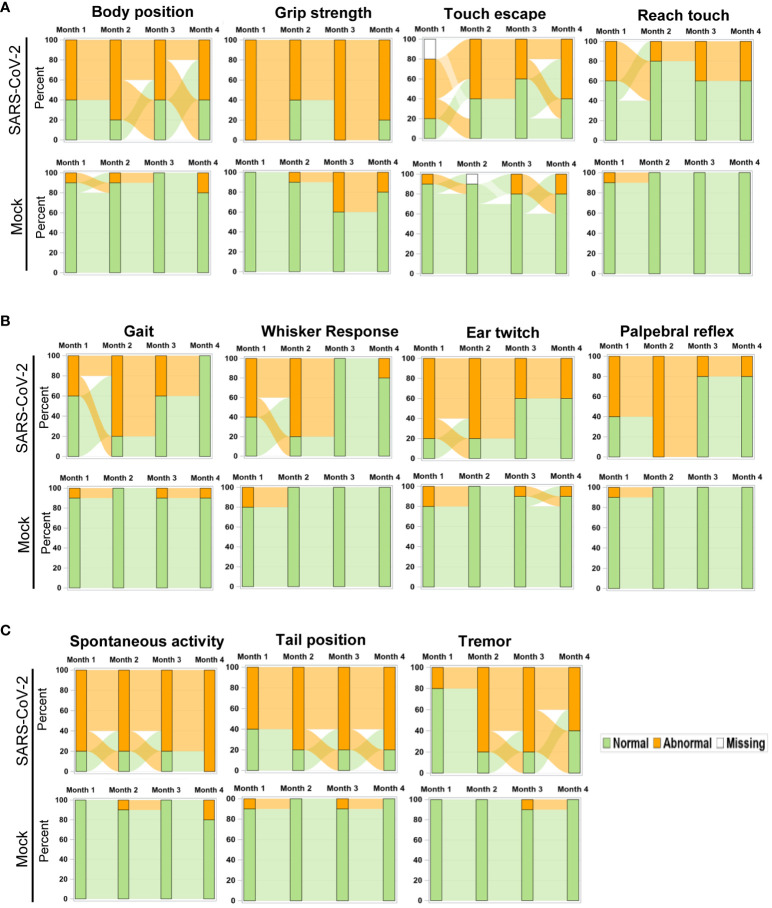
Longitudinal analysis of behavior changes 4 months post-acute infection in mice. Sankey bar charts were used to present results of SHIPA analysis. Functional status was defined as abnormal vs. normal and used to form the groups in the stacked bars at each time. Longitudinal stacked bar chart with Sankey-style overlays visualizes how the mice transit between abnormal and normal status over time. The *y*-axis is the percent in each group and the *x*-axis is the time for the measures collected. The link (bend) between the bars shows the transitions between two states over time. **(A)** Parameter abnormality patterns not changed within the 4-month pi period. **(B, C)** Parameter abnormality rates decreased **(B)** or increased **(C)** within the 4-month pi period.

To further assess the impact of SARS-CoV-2 infection on behavioral changes of surviving mice, SHIRPA analysis was performed longitudinally over the 4-month pi period. It was noted that in mice surviving Delta variant infection, the abnormal rates for the parameters such as body position, grip strength, touch escape, and reach touch remained unchanged during the 4-month period (**Figure 4A**). However, abnormal levels for gait, whisker response, ear twitch, and palpebral reflex decreased over time (**Figure 4B**). In contrast, abnormalities for parameters including spontaneous activity, tail position, and tremor increased over the 4-month period (**Figure 4C**). Overall, the neuropsychiatric state and motor behavior of Delta variant-infected mice remained impaired or even deteriorated months pi, whereas reflex and sensory functions appeared to recover over time.

Transcriptome analysis revealed persistent activation of immune pathways, and cognitive and neuronal dysfunction in the CNS months post-acute infection, though minimal microglia activation was observed. Potent Th1-prone cellular and antibody responses persisted in the periphery during post-acute infection.

**Figure 5:**
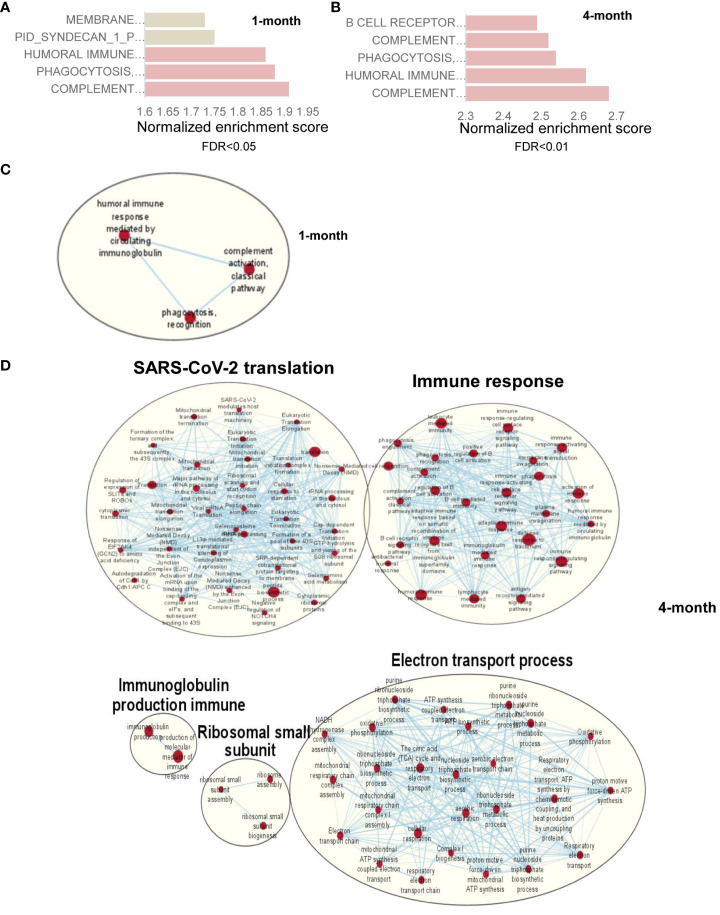
RNA-seq analysis of SARS-CoV-2- infected mouse brain shows genes with upregulation of immune signaling and enrichment of immune signaling-related pathways. **(A)** Pathway enrichment analysis of differential expressed genes using GSEA shows the top 5 upregulated pathways in SARS-CoV-2*-*infected mouse brain at 1 month **(A)** and 4 months **(B)** compared to mock-treated brain. Gray bar represents FDR >0.05 while orange bars represent FDR <0.05. **(C, D)** Cytoscape enrichment map (FDR *Q* value < 0.01) of GSEA pathways enriched in upregulated genes in SARS-CoV-2-infected mouse brain tissue at 1 month **(C)** and 4 months **(D)** pi compared to mock-treated sample. Clusters of nodes were labeled using the Auto Annotate feature of the Cytoscape application. Red nodes represent upregulated gene set enrichment and their node size represents the gene set size. The thickness of the line connecting the nodes represents the degree of overlap between two gene sets.

**Figure 6:**
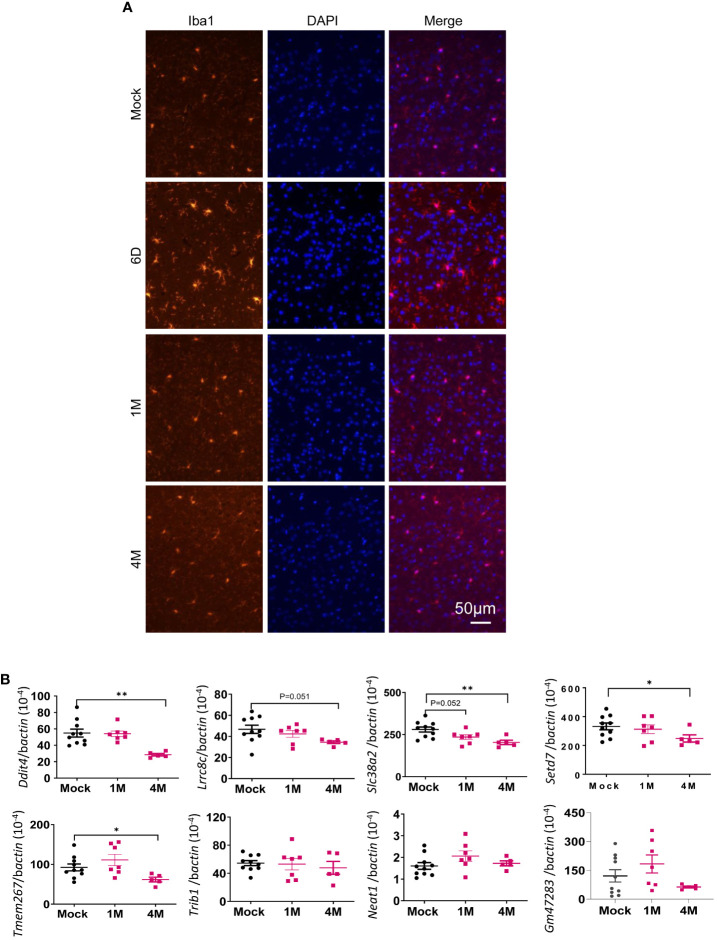
Microglia were activated during acute SARS-CoV-2 infection in the brain but were minimally activated post-acute infection. **(A)** Immunofluorescence images of brain tissues stained with DAPI (blue) and anti-Iba1 (red) at both acute infection (day 6, D6) and post-acute infection [1 month (M) and 4M pi]. **(B)** Q-PCR analysis of the levels of eight differentially expressed genes identified by transcriptomic analysis in the brain samples of mock- or SARS-CoV-2-infected samples at 1M and 4M. *n* = 5 to 10. **p < 0.01 or *p < 0.05, compared to the mock group.

To identify the host factors contributing to SARS-CoV-2 variant-induced PASC, we determined gene expression alterations by comparing the transcriptomes of mock and SARS-CoV-2 Delta variant-infected mouse brains using bulk RNA-seq. Transcriptome analysis identified 3,481 and 18 differentially expressed genes (FDR < 0.1) in 1-month and 4-month post-SARS-CoV-2 variant-infected mouse brains, respectively, compared to mock. We then performed GSEA to identify the biological processes that play a role in host response against SARS-CoV-2 infection. GSEA of RNA-seq data identified pathways related to immune signaling, such as the “complement activation pathway” and “phagocytosis recognition” (FDR < 0.01) as the top enriched pathways (**Figure 5A**, **Supplementary Figure 3A**, **Supplementary Table S2**). Interestingly, we also observed enrichment of the same immune signaling-related pathways “complement activation pathway,” “phagocytosis recognition,” and “humoral immune response mediated by circulating immunoglobulin” (FDR < 0.01) among the top five enriched pathways at 4-month pi (**Figure 5B**, **Supplementary Figure 3B**, **Supplementary Table S2**). Notably, the normalized enrichment scores (NESs) were higher for these pathways compared to the dataset at 1-month pi, an indication of persistence of immune activation in the CNS caused by infiltering immune cells and factors following the initial viral infection. Next, we utilized Cytoscape Enrichment Map and AutoAnnotate tools to identify biological networks that are associated with the enriched gene sets and found immune response cluster in both 1-month and 4-month pi datasets. In addition, other enriched gene sets were annotated as “SARS-CoV-2 translation,” “electron transport process,” and “ribosomal small subunit,” indicating that overall nervous system homeostasis is perturbed (**Figures 5C, D**). As microglia activation was often associated with cognitive changes and SARS-CoV-2 infection preferentially targets astrocytes (42–44), immunofluorescence staining was next performed to detect activation of microglia and astrocytes at both acute infection (day 6) and post-acute infection (1-month and 4-month pi). Microglia activation with increased cell processes was noted at day 6; however, minimal to mild activation of these cells were found at 1-month and 4- month pi (**Figure 6A**). Minimal astrocyte activation was detected at 1 month and 4 months pi (data not shown). These data suggest that infiltrating immune cells, not the residential immune cells, are involved in the immune pathway activation in the CNS at the post-acute stage. Q-PCR analysis was next utilized to determine eight differentially expressed genes identified by transcriptomic analysis. Reduced levels of expression of *Ddit4*, *Slc38a*2, *Tmem267m*, *Lrrc8c*, and *setd7* genes were noted at 4 months pi, which indicate ataxia telangiectasia neurodegenerative disease, impairment of memory, synaptic plasticity, motor, and cognitive abilities, neuronal dysfunction and degeneration, and cerebral ischemic stroke, respectively (45–48) (**Figure 6B**).

**Figure 7:**
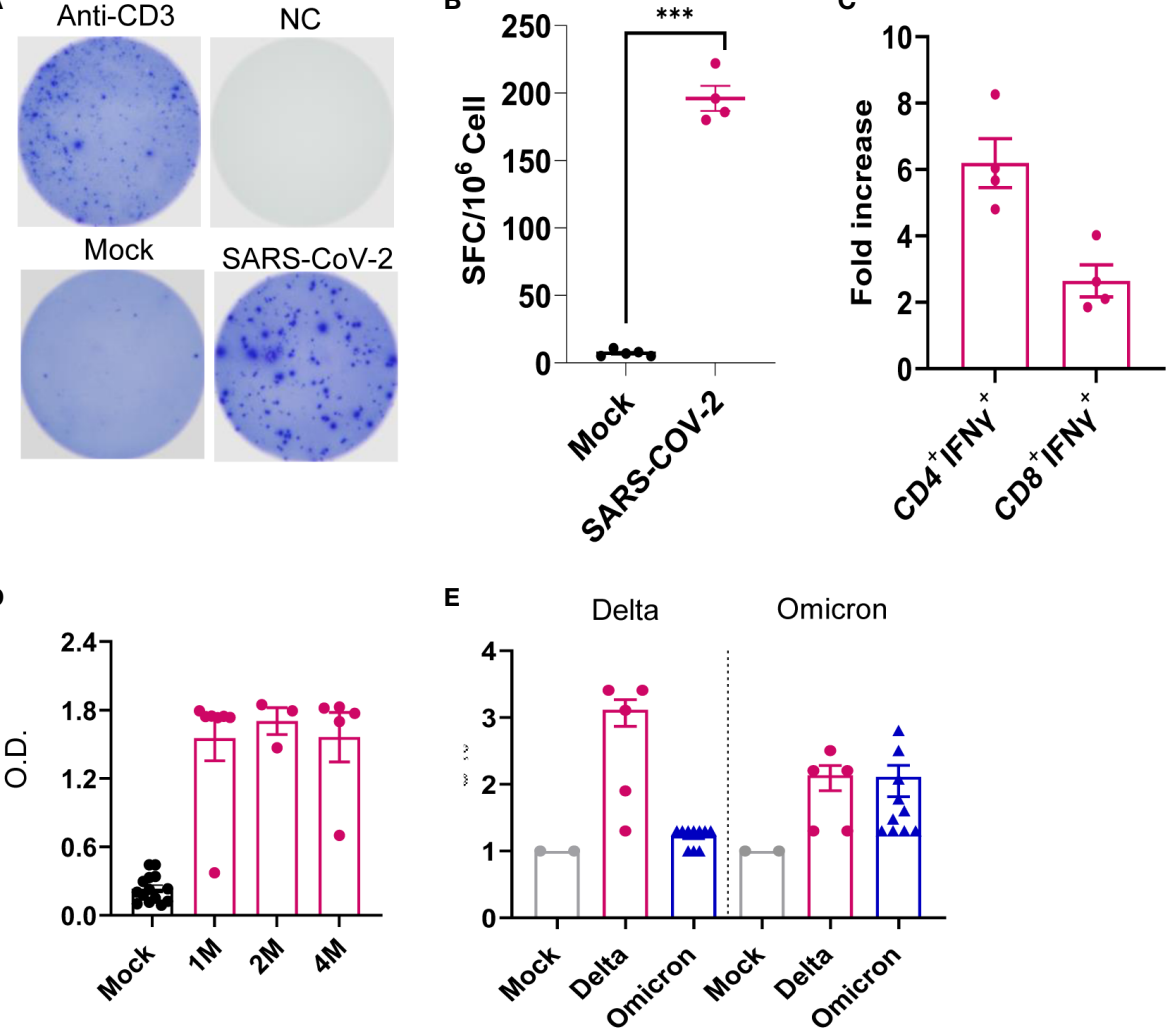
SARS-CoV-2 Delta variant induced persistent systemic cellular and humoral immune responses post-acute infection. K18-hACE2 mice were infected with a sublethal dose of SARS-CoV-2 Delta variant. **(A–C)** At 1 month pi, splenocytes were collected from surviving mice and mock group to measure T-cell responses. **(A, B)** ELISPOT quantification of splenic T-cell responses. Splenocytes were stimulated with SARS-CoV-2 S peptides, anti-CD3, or blank (negative control, NC) for 24 h. **(A)** Images of wells of T-cell culture. **(B)** Spot-forming cells (SFCs) were measured by IFN-γ ELISPOT. Data are shown as the number of SFCs per 10^6^ splenocytes. *n* = 5. **(C)** Splenocytes were cultured *ex vivo* with S peptide pools for 6 h, and stained for IFN-γ, CD3, CD4, or CD8. Fold increase of IFN-γ^+^ CD4^+^ and CD8^+^ T-cell expansion compared to the mock group is shown. **(D)** Sera of SARS-CoV-2 RBD-binding IgG titers at 1 month (M), 2M and 4M pi. O.D. values were measured by ELISA. *n* = 5 and 10 for Delta variant-infected and mock, respectively. **(E)** At 4M pi, sera neutralizing activity against SARS-CoV-2 Delta variant or Omicron B.A.2 variant was measured by plaque reduction neutralization test (PRNT). mNG-NT_50_ titers are shown, *n* = 2, 5, and 10 for mock-, Delta variant- or Omicron-infected group, respectively. ***p < 0.001, compared to the mock group.

We next assessed systemic immune responses in surviving mice post-acute infection. At 1 month pi, splenic T cells, including both CD4^+^ and CD8^+^ T-cell subsets, produced robust T helper (Th)-1 prone immune responses upon *in vitro* re-stimulation with S peptide pools compared to the mock group (**Figures 7A–C**). Furthermore, surviving mice also maintained high sera titers of RBD-binding IgG and neutralizing antibodies against Delta variant during the 4-month post-acute infection. Notably, similar levels of neutralization activity against Omicron strain were detected in the Delta variant-infected mice compared to mice surviving Omicron strain 4 months pi (**Figures 7D, E**). Overall, these results suggest that mice surviving SARS-CoV-2 Delta variant infection developed long-lasting Th1 and antibody responses in the periphery post-acute infection.

## 4 Discussion

The high risk of PASC is known to be associated with people with prior COVID-19 infection. The frequencies of PASC symptoms were reported to increase with SARS-CoV-2 variants, in particular, with those infected with the pre-Omicron variant compared to the original prototype virus infection (3, 35–37). Here, we infected K18 hACE2 mice with the SARS-CoV-2 Delta variant to recapitulate PASC in patients with COVID-19. We reported that the SARS-CoV-2 Delta variant replicated productively in lung and brain and triggered robust local inflammatory responses at acute infection in K18-hACE2 mice. Weight loss, neuroinflammation, and mortalities were observed during acute infection. Surviving mice showed viral clearance with no additional weight loss, and minimal neuroinflammation. However, persistent neuropsychiatric state and motor-associated behavior changes were observed in surviving mice for months post-acute infection.

Our longitudinal behavior studies indicate development of ataxia and cognitive dysfunction in SARS-CoV-2 variant-infected mice post-acute infection. The behavior studies also suggest that the neuropsychiatric state and motor behavior of surviving mice remain impaired or deteriorated for months pi, whereas reflex and sensory functions appear to recover over time. These findings align with a recent 2-year retrospective cohort study that reported an increased risk of psychotic disorder, cognitive deficit, dementia, and epilepsy or seizures persisted in long-COVID patients (49). Furthermore, the downregulation of expression levels of *Ddit4*, *Slc38a*2, *Tmem267m*, *Lrrc8c*, and *setd7* genes in the brain at 4 months pi is associated with ataxia, impairment of memory, synaptic plasticity, motor, and cognitive abilities, neuronal dysfunction and degeneration, and cerebral ischemic stroke (45–48). RNA-seq analysis of brain samples showed activation of several immune pathways including “complement activation pathway,” “phagocytosis recognition,” and “humoral immune response mediated by circulating immunoglobulin” at 1 month and 4 months pi. The transcriptome results support neurological behavior changes observed in the surviving mice. In addition, these findings suggest that infiltrating immune cells and circulating immune factors contribute to CNS disorder. For example, the complement-dependent engulfment of synapses may lead to the cognitive dysfunction. The spike protein and its fragment was reportedly to be able to cross the blood–brain barrier and enter the CNS (50), and this is directly involved in COVID-19-induced cognitive dysfunction (51) via complement-dependent engulfment of synapses in mice (52). In this study, no detectable levels of S1 in the CNS were found during the post-acute phase. We also noted neuroinflammation and microglia reactivity during acute infection, but minimal to mild microglia activation was found months post-acute infection. It is likely that PASC results from viral infection and/or viral fragment entry into the CNS and their associated neuroinflammation and neuronal injury during the acute infection stage. Interestingly, we also noted that surviving mice maintained potent protective systemic Th-1 prone and humoral immune responses post-acute infection. Combined with transcriptome results, these findings further suggest that systemic immune factors contribute to the development of PASC.

As reported earlier (24, 53), the K18-hACE2 mouse strain was generated by inserting multiple copies of the K18-hACE2 transgene on mouse chromosome 2. The K18-hACE2 transgene includes the K18 promoter, the first intron (with a mutation in the 3’ splice acceptor site to reduce exon skipping) from the human keratin 18 (*KRT18*) gene, a translational enhancer sequence from alfalfa mosaic virus, *hACE2*) coding sequence, exons 6–7, and the poly(A) signal of the human *K18* gene. The K18 promoter confers efficient transgene expression in airway epithelial cells and epithelia of internal organs, including the liver, kidney, and gastrointestinal tract. Because of its high expression of hACE2 in the lung and kidney tissues, we noted Delta variant-induced productive viral replication in these tissues during acute infection. Viral loads in liver were nevertheless barely detectable at both acute and chronic stages. Liver injury has been reported to develop as a post-COVID sequela (54, 55). The primary focus of this study is SARS-CoV-2 variant infection in the CNS tissues and its contribution to PASC pathogenesis. Future investigation will also focus on the impacts of SARS-CoV-2 infection on liver, kidney, and other peripheral tissues and understand the association with PASC.

Several rodent models have been used to study long-COVID (56–58). In line with a recent report in a golden hamster model of long-COVID, we found detectable infectious virus in the CNS during the acute infection phase, which is correlated with behavioral changes at 1 month after viral clearance (57). Induction of CCL11 expression in the periphery tissues and CNS during acute COVID-19 infection was reported in a mild-respiratory COVID model in immunocompetent mice via delivery of an AAV vector to express human ACE2 to the trachea and lungs (58). Furthermore, results from the transcriptome analysis align with the impaired neurogenesis findings reported in both studies. Nevertheless, we did not note microglia reactivity, neuroinflammation, and induction of proinflammatory cytokines in the CNS post-acute phase as reported in these studies. Both prior studies are limited to a shorter time post-acute infection (4 to 7 weeks pi) and the use of wild-type prototype virus. Another study also reported increased reactive astrocytes and microglia, hyperphosphorylated TDP-43, and tau, and a decrease in synaptic protein synaptophysin-1 and defective neuronal integrity in A/J mice 12 months post-infection. Although the study recapitulates long-term sequelae of COVID-19, a mouse hepatitis virus 1 (MHV-1) was used in this study (59, 60).

In summary, our results suggest that infection in K18-hACE2 mice recapitulates the persistent clinical symptoms reported in long-COVID patients. Our immunological and transcriptomic analysis provides new insights into the pathogenesis of the disease. Given that the formation and consolidation of learning and memory occurs primarily within the hippocampus region of the brain and alteration of hippocampal cells in clinical and preclinical model post SARS-CoV-2 infection along with its connection with the olfactory dysfunction worsen cognition, additional studies in K18-hACE2 mice for the evaluation of structure and function of hippocampus are needed (61, 62). Moreover, development of dysautonomia, a nervous system disorder that disrupts autonomic body processes, was reported to be the most recurrent type of neurological disorder post-COVID and has been linked to the neuro-psychological sequelae of long-COVID, for instance, cognitive impairment (63). Future investigation of autonomic neuropathy in the preclinical model is likely to provide novel insights into SARS-CoV-2-induced PASC. Overall, the K18-hACE model of long-COVID may be useful to evaluate efficacy for the future development of novel SARS-CoV-2 vaccines or therapeutics.

## Data availability statement

The datasets presented in this study can be found in online repositories. The names of the repository/repositories and accession number(s) can be found below: GSE260625 (GEO).

## Ethics statement

The animal study was approved by Institutional Animal Care and Use Committee, University of Texas Medical Branch, Galveston, TX, USA. The study was conducted in accordance with the local legislation and institutional requirements.

## Author contributions

AS: Conceptualization, Data curation, Formal analysis, Methodology, Visualization, Writing – review & editing, Investigation, Validation. AA: Conceptualization, Data curation, Formal analysis, Investigation, Visualization, Writing – review & editing, Validation. A: Data curation, Formal analysis, Investigation, Writing – review & editing, Methodology, Software. B-HP: Formal analysis, Investigation, Methodology, Writing – review & editing. XY: Data curation, Formal analysis, Methodology, Software, Writing – review & editing. JZ: Data curation, Formal analysis, Investigation, Methodology, Writing – review & editing. VK: Data curation, Formal analysis, Investigation, Validation, Visualization, Writing – review & editing. PK: Conceptualization, Funding acquisition, Resources, Writing – review & editing. WJ: Data curation, Methodology, Software, Writing – review & editing. P-YS: Conceptualization, Methodology, Writing – review & editing. PS: Data curation, Formal analysis, Investigation, Methodology, Software, Writing – review & editing. IC: Investigation, Methodology, Software, Writing – review & editing, Conceptualization, Resources. TW: Methodology, Resources, Writing – review & editing, Conceptualization, Data curation, Formal analysis, Funding acquisition, Project administration, Supervision, Visualization, Writing – original draft.
