# Supplementary figures and images for: A murine model of post-acute neurological sequelae following SARS-CoV-2 variant infection
Source: Front Immunol. 2024 May 3;15:1384516. doi: 10.3389/fimmu.2024.1384516 (PMC11099216; doi:10.3389/fimmu.2024.1384516)

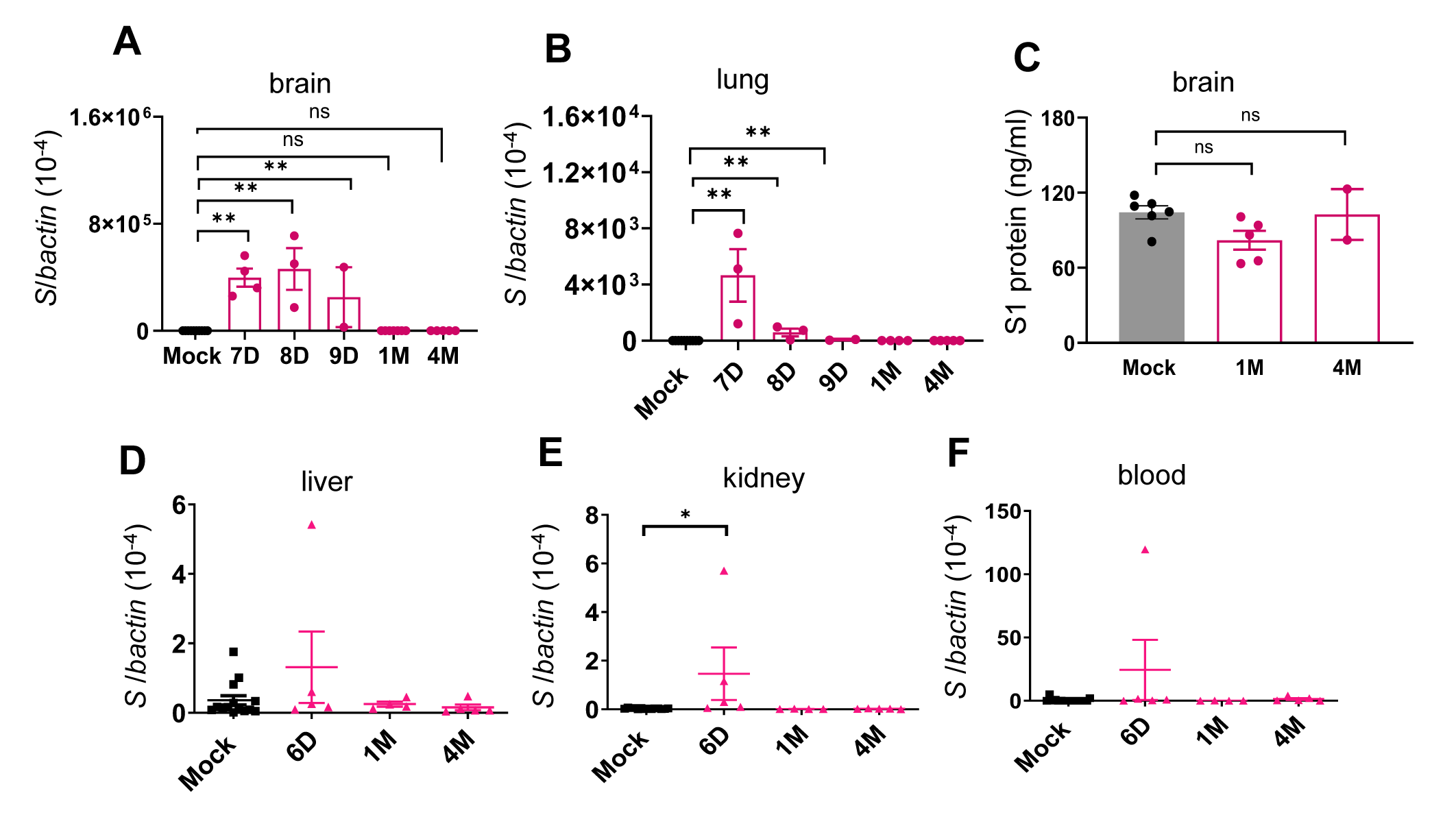

Supplement: Supplementary file 1 [file Image_1.tif]

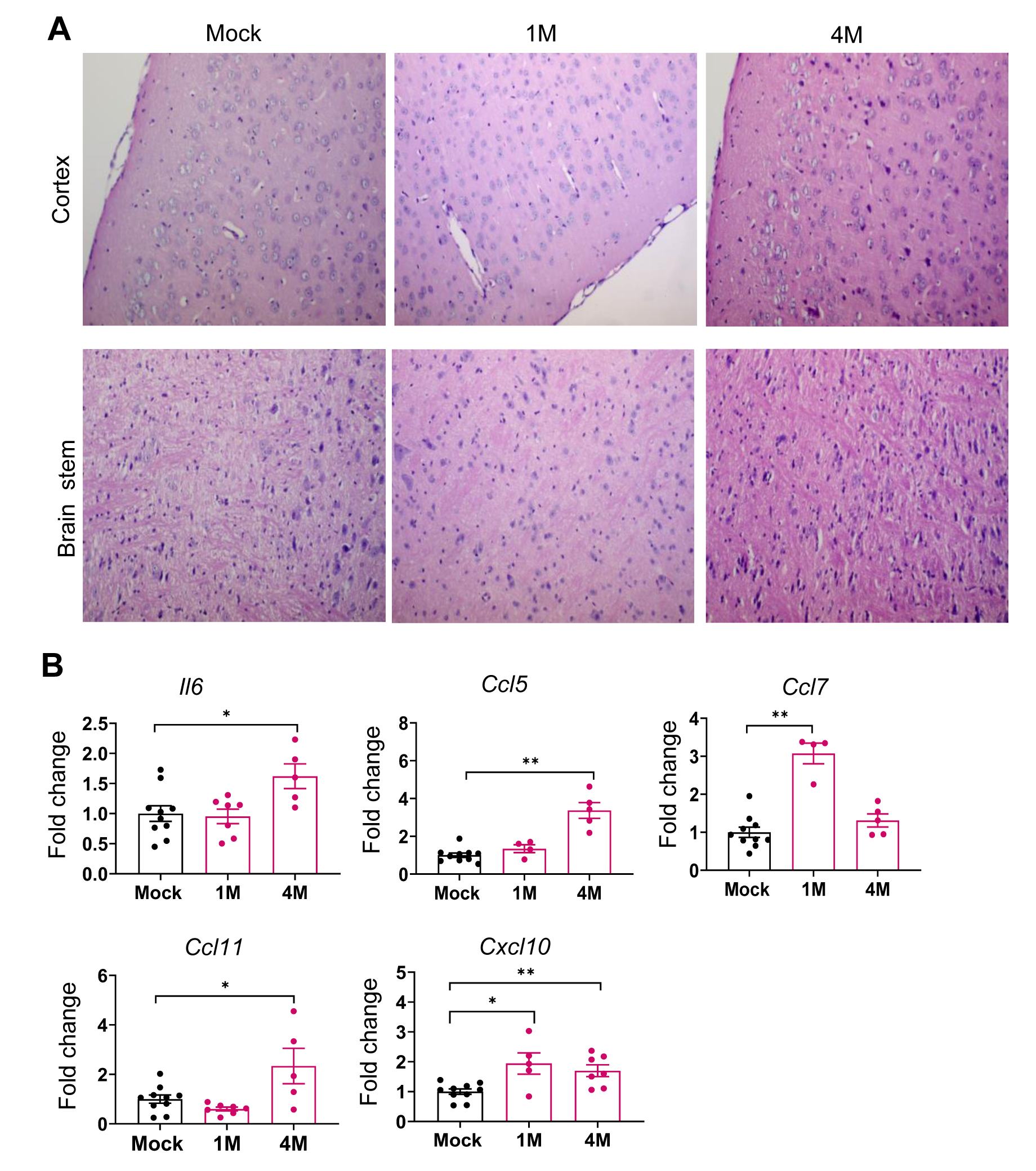

Supplement: Supplementary file 2 [file Image_2.tif]

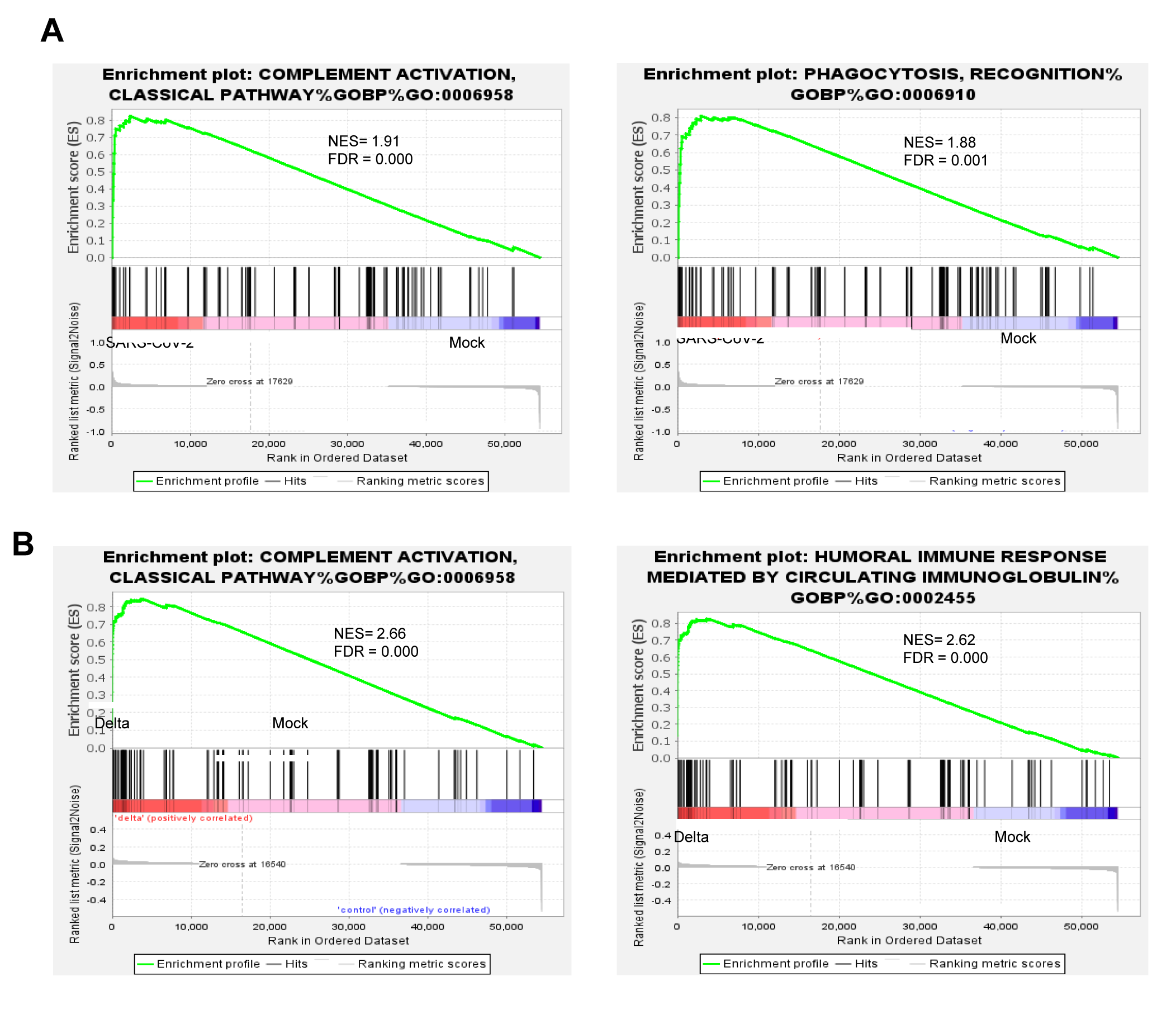

Supplement: Supplementary file 3 [file Image_3.tif]
