# Supplementary material for: A murine model of post-acute neurological sequelae following SARS-CoV-2 variant infection
Source: Front Immunol. 2024 May 3;15:1384516. doi: 10.3389/fimmu.2024.1384516 (PMC11099216; doi:10.3389/fimmu.2024.1384516)
Supplement: Supplementary file 4 [file Table_1.docx]

**Supplementary Figure Legends:**

**Supplementary Fig 1. Viral loads in brain and periphery tissues at acute and post-acute infection.** K18-hACE2 mice were infected with a sublethal dose of SARS-CoV-2 Delta variant, or PBS (mock). Viral loads in brain (**A**), lung (**B**), liver (**D**), kidney (**E**), and blood (**F**) tissues at indicated days (D) or months (M) pi were measured by Q-PCR. **C.** S1 protein levels in brain lysates were measured by ELISA. Data are presented as means ± SEM. ****P* < 0.001, ***P* < 0.01, or **P* < 0.05 compared to mock.

**Supplementary Fig 2. SARS-CoV-2 Delta variant infection induces minimal or mild inflammation in the brain and lung post-acute infection. A.** Histopathology of Delta variant- infected or mock-infected brain Cortex and brain stem at 1 month (M) or 4M pi. Views of representative areas from each group show no inflammation is observed in the control or infected brains. Bar = 80 μm. **B.** Cytokine and chemokines expression levels in the lungs at 1M and 4M were measured by Q-PCR. Data are presented as the fold increase compared to mock-infected mice (means ± SEM). n= 5 to 10. ***P* < 0.01, or **P* < 0.05 compared to mock.

**Supplementary Fig 3. RNA-seq analysis of SARS-CoV-2- infected mouse brain shows genes with upregulation of immune signaling and enrichment of immune signaling related pathways.** Representative GSEA plots showing enrichment of immune pathways based on RNAseq results of brain samples collected from 1-month (**A**) and 4-month (**B**).

**Supplementary Table 1: Sequence of qPCR primers: for validation of RNAseq analysis**

| **Gene** | **Primer sequence** | **Reference** |
| --- | --- | --- |
| *Neat1* | Forward: GTTCCGTGCTTCCTCTTCTG  Reverse: GTGTCCTCCGACTTTACCAG | ^1^ |
| *Lrrc8c* | Forward: AACTCGGTCACCGGAATCAT  Reverse: CCCCAGAGATTAATGTGGCT | ^2^ |
| *Trib1* | Forward: GGAAGTTCGTCTTCTCCACCGA  Reverse: GCAGCCATGTTTATCTGACAGCG | <https://www.origene.com/catalog/gene-expression/qpcr-primer-pairs/mp217531/trib1-mouse-qpcr-primer-pair-nm_144549>  NCBI Reference Sequence:  NM_144549.4 |
| *Slc38a2* | Forward: TAATCTGAG CAATGCGATTGTGG  Reverse: AGATGGACGGAGTATAGCGAAAA | ^3^ |
| *Tmem267* | Forward: GGCAGTAGTCACTGGAATCAGG  Reverse: CTTCGCGGAAGAGTCAAAGCGG | <https://www.origene.com/catalog/gene-expression/qpcr-primer-pairs/mp211555/tmem267-mouse-qpcr-primer-pair-nm_001039244>  NCBI Reference Sequence: NM_001039244.4 |
| *Setd7* | Forward: TTGACGGAGAGATGCTCGAAGG  Reverse: GAAGGAGAGCATCGCTGGAGAT | <https://www.origene.com/catalog/gene-expression/qpcr-primer-pairs/mp215317/setd7-mouse-qpcr-primer-pair-nm_080793>  NCBI Reference Sequence: NM_080793.6 |
| *Ddit4* | Forward: ACTGCGAGTCCCTGGACAGCA  Reverse: TTGGCACACAGGTGCTCATCCT | <https://www.origene.com/catalog/gene-expression/qpcr-primer-pairs/mp203836/ddit4-mouse-qpcr-primer-pair-nm_029083>  NCBI Reference Sequence: NM_029083.2 |
| *Gm47283* | Forward: CTGAGAAGAGGGCGTCAGAT  Reverse: CTGTCAGAGTGAAGGGCAGA | ^4^ |

**Supplementary References:**

1. Barry, G., Briggs, J.A., Hwang, D.W., Nayler, S.P., Fortuna, P.R., Jonkhout, N., Dachet, F., Maag, J.L., Mestdagh, P., Singh, E.M., et al. (2017). The long non-coding RNA NEAT1 is responsive to neuronal activity and is associated with hyperexcitability states. Sci Rep *7*, 40127. 10.1038/srep40127.

2. Concepcion, A.R., Wagner, L.E., 2nd, Zhu, J., Tao, A.Y., Yang, J., Khodadadi-Jamayran, A., Wang, Y.H., Liu, M., Rose, R.E., Jones, D.R., et al. (2022). The volume-regulated anion channel LRRC8C suppresses T cell function by regulating cyclic dinucleotide transport and STING-p53 signaling. Nat Immunol *23*, 287-302. 10.1038/s41590-021-01105-x.

3. Du, C., Xu, H., Cao, C., Cao, J., Zhang, Y., Zhang, C., Qiao, R., Ming, W., Li, Y., Ren, H., et al. (2023). Neutral amino acid transporter SLC38A2 protects renal medulla from hyperosmolarity-induced ferroptosis. Elife *12*. 10.7554/eLife.80647.

4. Gao, F., Zhao, Y., Zhang, B., Xiao, C., Sun, Z., Gao, Y., and Dou, X. (2022). Suppression of lncRNA Gm47283 attenuates myocardial infarction via miR-706/ Ptgs2/ferroptosis axis. Bioengineered *13*, 10786-10802. 10.1080/21655979.2022.2065743.
